# Sampling 'hard-to-reach' populations in health research: yield from a study targeting Americans living in Canada

**DOI:** 10.1186/1471-2288-8-57

**Published:** 2008-08-18

**Authors:** Danielle A Southern, Steven Lewis, Colleen J Maxwell, James R Dunn, Tom W Noseworthy, Gail Corbett, Karen Thomas, William A Ghali

**Affiliations:** 1 Centre for Health and Policy Studies & Dept. of Community Health Sciences, University of Calgary, 2500 University Drive NW, Calgary, Alberta, T2N 1N4, Canada; 2 Access Consulting Ltd, Access Consulting Ltd., 211-4th Ave. S, Saskatoon SK S7K 1N1, Canada; 3 Department of Medicine, University of Calgary, 2500 University Drive NW, Calgary, Alberta, T2N 1N4, Canada; 4 Centre for Research on Inner City Health, St. Michael's Hospital, Toronto, Canada; 5 Department of Geography, University of Toronto, Ontario M5S 1A8, Canada; 6 Office of Communications, Faculty of Medicine, University of Calgary, 2500 University Drive NW, Calgary, Alberta, T2N 1N4, Canada

## Abstract

**Background:**

Some populations targeted in survey research can be hard to reach, either because of lack of contact information, or non-existent databases to inform sampling. Here, we present a methodological "case-report" of the yield of a multi-step survey study assessing views on health care among American emigres to Canada, a hard-to-reach population.

**Methods:**

To sample this hard-to-reach population, we held a live media conference, supplemented by a nation-wide media release announcing the study. We prepared an 'op-ed' piece describing the study and how to participate. We paid for advertisements in 6 newspapers. We sent the survey information to targeted organizations. And lastly, we asked those who completed the web survey to send the information to others. We use descriptive statistics to document the method's yield.

**Results:**

The combined media strategies led to 4 television news interviews, 10 newspaper stories, 1 editorial and 2 radio interviews. 458 unique individuals accessed the on-line survey, among whom 310 eligible subjects provided responses to the key study questions. Fifty-six percent reported that they became aware of the survey via media outlets, 26% by word of mouth, and 9% through both the media and word of mouth.

**Conclusion:**

Our multi-step communication method yielded a sufficient sample of Americans living in Canada. This combination of paid and unpaid media exposure can be considered by others as a unique methodological approach to identifying and sampling hard-to-reach populations.

## Background

There are many challenges associated with conducting research on 'hard-to-reach' populations, beginning with how to identify and sample certain groups of individuals for health research [1-5]. Some populations are particularly vulnerable and hard-to-reach, including the homeless [2]. Other populations, however, may be defined by characteristics such as ethnicity or country of origin that may not be recorded in routinely available data sources [6]. Such was the challenge in a study to obtain the views of Americans living in Canada on their experiences of health care in Canada and the United States [7].

Americans in Canada are a hard-to-reach group. Immigration records exist but because of privacy restrictions, there is no readily-available database that researchers may access to identify, and subsequently contact, émigrés by country of origin. A further complication in our case was the desire to obtain a representative sample of recent (2–5 years) American arrivals in Canada.

Faced with this constraint, we devised an alternative multi-step approach to obtaining subjects for our research by informing the public of our study through a combination of a media release, an 'op-ed' (opposite the editorial page) submission, and an ensuing sequence of paid advertisements in major Canadian newpapers (see Methods section). At the outset, we had no idea of how many subjects our method would yield, and as such, one of our research questions was, in fact, to assess the yield of such an approach. The main results of our study are published elsewhere [7]. Here we present a detailed report of the methods used as well as its yield. The success of this combined approach would depend on two outcomes: the extent to which the media responded to the media event, and the yield of potential study subjects over time in response to the media release and subsequent advertisements. The insights gained from our experience may be of value to researchers conducting research on such hard-to-reach populations.

## Methods

The main objective of the Americans in Canada study was to report the experiences and views of Americans living in Canada who have used both health care systems as adults. We developed and pre-tested a web-based survey instrument to gather information on respondents' demographics, reasons for moving to Canada, health status, use and personal costs of health care, assessments of the timeliness and quality of care in several categories in both countries, and overall system preferences. We defined the "ideal" respondents as Americans with at least 2 years experience as adults in the US system responsible for their own health insurance (i.e., not covered under a parent's insurance), and who had been living in Canada for at least two but no more than five years, to ensure reasonable recall of experiences in both systems. Given the unavailability of a database of Americans residing in Canada, we designed an exploratory study using a novel method for obtaining a sample of respondents.

### Recruitment goals and strategies

Study recruitment was targeted at American émigrés with the characteristics described above. We set an *a priori *target of at least 200 respondents to achieve a margin of error of +- 7 percent (95% CI) for responses.

We used five techniques to solicit responses. First, through the offices of the University of Calgary, Faculty of Medicine Office of Communications we held a live media conference, supplemented by a nation-wide media release . The media event included short presentations by one of the study investigators as well as two émigrés to Canada who recounted their experiences of health care in both countries. The media release announced the study, highlighted its importance, and informed respondents how to participate. The intent was to reach as many electronic and print media as possible at virtually no cost. The premise was that a head-to-head comparison of health care systems was potentially newsworthy in itself, and would create media exposure that would generate respondents. Second, one month after the media conference, we prepared and nationally distributed an op-ed piece that outlined the purpose of the study, why it was unique and important, and how to participate. The intent was to reinforce the early exposure and reach new audiences. Third, we advertised the study in six newspapers in 3 cities: Toronto, (the *Globe and Mail *and *National Post*); Calgary (the *Herald *and the *Sun*; and Vancouver (the *Province *and the *Sun*). The Toronto papers have a national as well as a local circulation. The three cities account for 31 percent (79 000) of the 258 000 residents who moved to Canada from the US and about 40 percent of recent arrivals [8]. The intent was to guarantee exposure in important media outlets likely to be read by the eligible population. Fourth, we sent the survey information and coordinates to individuals and groups likely to be eligible to participate, i.e., American consulates, Democrats in Canada, and Republicans in Canada, and asked them either to respond as individuals, or forward the survey information to their membership or contact lists. And fifth, we asked those who had logged onto the survey site to send the information to others likely to meet the eligibility criteria.

### Ethical approval

Ethics approval was granted from the Health Research Ethics Board at the University of Calgary.

The survey was posted on the web on April 6, 2005 and remained "live" until July 31, 2005. We installed a toll-free telephone number, accessible from April 8, 2005 to August 31, 2005, to handle inquiries and provide technical assistance. The phone logged 30 calls with 12 requests for mail-out surveys. We chose a web-based survey because we assumed that a very high percentage of the target audience would be connected to the internet, and also because respondents might be more willing to answer potentially sensitive questions anonymously in electronic format rather than in personal interviews. It was also far less costly. We were able to record the internet protocol (IP) address of the computer terminal of each respondent to reduce the likelihood of multiple entries by the same person. Once duplicate IP addresses were flagged, they were discarded to ensure anonymity.

### Key measures

We tracked the media coverage from the date of our press release (April 7, 2005) until the end of July, 2005, using a media surveillance service provided by Bowdens Media (Toronto, Ontario). For yield of survey respondents, meanwhile, we tracked the number of subjects having accessed the survey on the website as well as the number of completed responses to the survey by month. As part of our survey, we asked individuals to indicate which communication strategy was responsible for them finding our survey. The possible responses included newspaper, radio, TV, advertising, internet and word of mouth.

### Data analysis

Our data analysis and presentation were primarily descriptive. We graphically present the number of individuals accessing the survey and completing the survey by day, from the time the study began until the survey was closed. We also categorically report the characteristics of respondents, as well as the information source(s) that led respondents to complete the survey.

## Results

### Media Response to the initial press release

The nationally-distributed press release for our study was successful in attracting considerable media attention, as it led to 4 television news interviews, 10 newspaper stories, one newspaper editorial, and 2 radio interviews (the latter of which were repeated for a total of 9 broadcasts). During all interviews and newspaper stories, either the toll free number or the web site address were advertised (an important element for our survey method to succeed). The stories appeared in the media outlets of 13 different cities.

### Yield of study subjects

The media exposure in response to the initial event and release occurred on or soon after April 7, 2005. This was then followed by the subsequent publication of our op-ed piece on May 11, 2005, and a series of paid advertisements in newspapers appearing from May 28, 2005 to June 4, 2005. Our *a priori *recruitment goal of 200 study participants was reached at the two-month mark. Eventually, 458 unique individuals accessed the on-line study survey.

**Figure 1:**
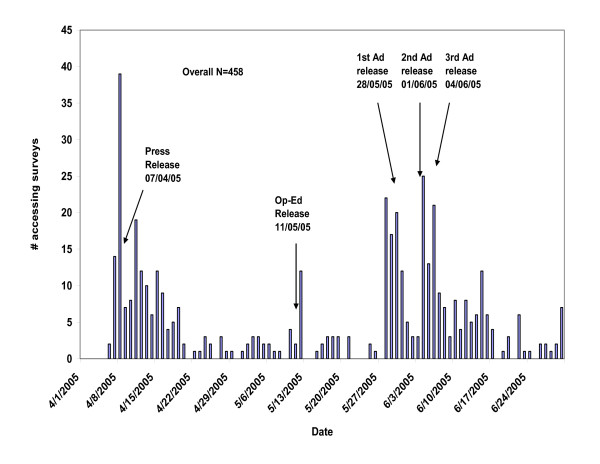
Distribution of accessed online surveys during the recruitment period of April 7, 2005 – July 31, 2005 (n = 458 accessed surveys).

Figure 1 shows the distribution of individuals accessing the survey, by week. The figure demonstrates that our initial live media event supplemented by a nation-wide press release was successful in attracting people to access our survey and at least read its content, averaging about 10 per day for approximately two weeks, and tailing off considerably thereafter. The op-ed piece led to a small (but non-sustained) increase in the number accessing the survey, with return to a low-level of survey visits. The paid advertisements in late May-early June then increased the daily access rate to approximately 11 that, like the initial press release, persisted for approximately 2 weeks before subsiding.

**Figure 2:**
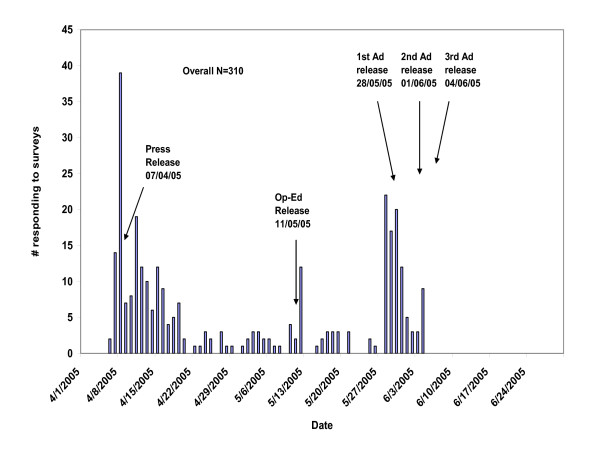
Distribution of responses to online survey during the recruitment period of April 7, 2005.

We do not know if all of the 458 individuals accessing the survey were eligible to complete it according to our stated eligibility criteria. However, 387 (84.5 percent) went on to complete part of the survey, after indicating that they considered themselves eligible for the study. Our study's focus was on health care utilization and only 310 individuals (i.e., 310 of the 458 [67.7 percent] accessing the survey) provided useable information for our study; these 310 individuals thus constituted our final study population. Figure 2 shows the distribution of useable responses by week among these 310 individuals, with a general pattern that resembles that for the larger pool of 458 individuals who accessed the survey. There were no eligible responses after June 5 (Figure 2), despite some individuals accessing the survey after that date (Figure 1).

### Characteristics of respondents

The 310 respondents ranged from 22 to 90 years of age (median = 47 years), with 43.0 percent males. As expected for American immigrants to Canada as a whole, the respondents generally were of high socioeconomic status, with 84.6 percent having attained a university degree or higher, and with only 14.8 percent having household incomes less than $50 000; 13.8 percent reported household incomes greater than $200000. This is in contrast to the general population in Canada, that has a median age of 37.6 years, 51 percent males, 15 percent having attained a university degree or higher, 44 percent with household incomes less than $50 000, and only 5 percent with household incomes greater than $200 000 [6].

### Origin of information

All but one respondent indicated the source of information that led them to access the survey (Table 1). These respondents most frequently mentioned newspaper stories (17.7 percent) followed by newspaper advertisements (14.2 percent) and friends/family (12.3 percent) as their information source. Eighty-six (28.6 percent) reported that a combination of sources triggered their attempt to complete the online survey.

**Table 1:** Sources leading to full completion of survey (n* = 310)

**Source**	**N (%)**
Media	120 (38.7)
Newspaper story	55 (17.7)
Newspaper Advertisement	44 (14.2)
TV interview	13 (4.2)
Radio interview	8 (2.6)
Word of mouth	75 (24.9)
Friends/family	38 (12.3)
Work colleagues	21 (6.8)
Internet surfing	16 (5.2)
Any Combination of media/word of mouth	86 (28.6)
Not sure/unknown	28 (9.0)

* n = total number of participants with complete data to our question on source of information about the survey.

Learning of the survey through a newspaper story and/or by watching TV was less likely among those with a Master's degree or higher than those with an undergraduate degree or less (11.4 percent vs. 25.9 percent, p = < 0.01 for newspaper and 2.3 percent vs. 6.7 percent, p = 0.05 for watching TV). Those with a Master's degree or higher were more likely to have learned of the survey from a newspaper advertisement or through friends or family than those with an undergraduate degree or less (18.9 percent vs. 8.2 percent, p < 0.01 for advertisements and 15.4 percent vs. 8.2 percent for friends or family). Surfing the Internet was more often the source of information for males than it was for females (8.3 percent vs. 2.8 percent, p = 0.03).

## Discussion

This methodological "case-report" presents our multi-method approach to recruiting a hard-to-reach study population for a survey study. The method used was generally successful in recruiting respondents to our survey of the health care views of Americans living in Canada. Total study costs were low at only $28,000 Canadian, the bulk of which paid for the newspaper ads.

Whether the method would achieve similar success in another context is likely dependent on the nature of the hard-to-reach population and the topic. By virtue of the regulations governing immigration to Canada, Americans in Canada are a prosperous, well-educated, and highly literate population. The study topic–health care–is of wide general interest in both Canada and the US. An added dimension was the uniqueness of the approach: to our knowledge, ours is the first study asking one group of respondents to compare two health care systems experienced first-hand. This combination of features likely made the study more newsworthy. Researchers studying other populations and topics may or may not succeed in generating such media interest.

All of the study investigators were from the same institution; had we assembled collaborators across the country and orchestrated a simultaneous series of live media events to launch the study, the response rate may well have increased (along with complexity and costs). Despite these caveats, the low cost and relatively high yield of our method may provide an option for researchers facing the challenge of accessing certain hard-to-reach populations. Another limitation to the study is that there was no way to ensure that participants did not participate more than once. We were able to monitor multiple entries by scanning for duplicated IP addresses. However, if a given participant chose to complete the survey a second time and was using another IP address when doing so, we would have no way to detect this.

As is the case with all self-reported surveys, it is possible that participants did not provide legitimate answers [9]. Any survey study also needs to consider non-responders in a targeted population and the extent to which they may differ from responders. In our case, there could be bias relating to education, higher SES, education, and general level of 'media awareness' (that would have almost certainly been higher in those who responded to our survey).

Our strategy, in broad terms, included a combination of three main elements: 1) a media event and press release that generated media attention, 2) paid advertisements, and 3) a 'snow-ball' approach of asking respondents to notify their acquaintances of the study. The patterns demonstrated in Figure 1 confirm that each of these elements contributed to our search for subjects. The press release/media coverage and the later paid advertising seem to have yielded approximately equal numbers of subjects over a similar two to three week time period. Given that the former cost a small fraction of the latter, researchers contemplating future studies may want to pursue the 'newsworthiness' potential of their subjects. It also appears as though there was a persisting trickle of responses that continued independent of either the media coverage or our advertisements. We speculate that this may have been the result of our snowball method of encouraging respondents to notify their acquaintances of our study. Approximately 25–30 percent of our overall survey responses came at non-peak enrollment times, when the snowball effect may have been at play.

In our case, it is also notable that the news release strategy and paid advertisements contributed approximately equally to our study's overall subject yield, with the research cost obviously being lower for the press release than it was for our paid advertisements. This may be of relevance to researchers in terms of anticipating project budgets.

Even where a topic is newsworthy, media exposure may depend on the other stories competing for attention at the time. This phenomenon may have influenced our study yield somewhat, as our media conference and press release were held on April 7, 2005, just five days after the death of Pope John Paul II, while media coverage of the aftermath of his death was still extensive. Our release also coincided with the lifting of a publication ban relating to a Canadian political scandal regarding irregularities in Federal government payments to advertising agencies in Quebec. Our newspaper opinion-editorial piece of May 11, 2005 was similarly confounded by competing news, as it appeared the day following a highly publicized non-confidence vote in the Canadian House of Commons. We can only speculate on the potential effect that these competing stories had on our goal of recruiting study subjects; it is possible that yield might have been somewhat greater had our study-related media coverage occurred in quieter news periods.

Similarly, the impact and yield of paid advertisements may vary according to seasonal or timing factors (e.g., long weekends) that may affect readership numbers. In our case, we targeted major Canadian newspapers for advertisements on days where the circulation is highest. Such an approach is likely to produce the highest yield, but also costs somewhat more.

While we have declared our method as a success on the grounds that we obtained a large enough number of responses from a hard-to-reach population to generate analyzable responses to a fairly extensive survey, the limitations are also apparent. The final sample was skewed towards residents of Alberta–home of the investigators' institution and site of the media event–and those with high socio-economic status. We were able to control for these characteristics in the analysis, and they proved to be minor influences on respondents' views. The final number of fully completed surveys was 310, which was well above our minimum target of 200, but short of the number required for more detailed statistical analyses. Again, though, the study costs were modest, and this was the first attempt at the method [7].

## Conclusion

A combination of paid and unpaid exposure in media outlets can in at least some circumstances yield a significant number of responses from identifiable but hard-to-reach populations to a web-based survey. Other researchers may want to consider some or all elements of the methods used in our study in other contexts, bearing in mind that feasibility, costs, and yield will vary according to the nature of the population and the topic.

## Competing interests

The authors declare that they have no competing interests.

## Authors' contributions

DAS performed the analysis and drafted the manuscript. SL and WAG participated in the design of the study and in the drafting of the manuscript. CJM, JRD, TWN, GC and KT participated in the design of the study. KT and GC participated in the advertising and dissemination of study information. All authors read and approved the final manuscript.

## Pre-publication history

The pre-publication history for this paper can be accessed here:


